# Supplementary material for: Using Functional Annotations to Study Pairwise Interactions in Urinary Tract Infection Communities
Source: Genes (Basel). 2021 Aug 6;12(8):1221. doi: 10.3390/genes12081221 (PMC8393552; doi:10.3390/genes12081221)
Supplement: Supplementary file 1 [file genes-12-01221-s001.zip › main.pdf]

Using Functional Annotations to Study Pairwise Interactions in Urinary Rract Infection Communities

Supplementary Tables and Figures

Table S1. Table containing the selected genomes for the *in silico* UTI community. A list of strains was made similar as in the experimental community. Genomes were selected from NCBI database following an order of preference by being related to urine or UTI, human isolate or lab strain, animal isolate and, finally, any other complete genome available. The information was retrieved in September 2017. U.: Uropathogenic. E.i.H.r: Environment isolate. Highly resistant. R.: representative.

Genus	**Group**	**Strain**	**Accession number**	**Origin**
Escherichia	Ecoli	E.coli cft073	nc_004431.1	UPEC (U. *E. coli*)
Escherichia	Ecoli	E. coli 536	nc_008253.1	UPEC,
Escherichia	Ecoli	E. coli IAI39	nc_011750.1	UPEC
Escherichia	Ecoli	E. coli UTI89	nc_007946.1	UTI isolate
Escherichia	Ecoli	E. coli E24377A	nc_009801.1	Human isolate
Escherichia	Ecoli	E. coli HS	nc_009800.1	Human isolate
Escherichia	Ecoli	E. coli O83-H1-NRG857C	nc_017634.1	Human isolate
Escherichia	Ecoli	E. coli O111-H-11128	nc_013364.1	Human isolate
Escherichia	Ecoli	E. coli O157-H7-Sakai	nc_002695.1	Human isolate
Escherichia	Ecoli	E. coli SE11DNA	nc_011415.1	Human isolate
Escherichia	Ecoli	E. coli O103-H2-12009	nc_013353.1	Human isolate
Escherichia	Ecoli	E. coli O104-H4-2011C3493	nc_018658.1	Human isolate
Escherichia	Ecoli	E. coli SE15DNA	nc_013654.1	Human isolate
Escherichia	Ecoli	E. coli BL21-DE3	nc_012892.2	Lab strain
Escherichia	Ecoli	E. coli BW2952	nc_012759.1	Lab strain
Escherichia	Ecoli	E. coli ATCC8739	nc_010468.1	Lab strain
Escherichia	Ecoli	E. coli K12-DH10B	nc_010473.1	Lab strain
Escherichia	Ecoli	E. coli K12-W3110DNA	nc_007779.1	Lab strain
Escherichia	Ecoli	E. coli K12-MG1655	nc_000913.3	Common isolate
Escherichia	Ecoli	E. coli SMS-3-5	nc_010498.1	E.i.H.r
Enterococcus	Ent	E. faecalis fdaargos338	cp_022059.1	Urine isolate
Enterococcus	Ent	E. faecium Aus004	nc_017022.1	Human isolate
Enterococcus	Ent	E. faecium Aus0085	nc_021994.1	Human isolate
Enterococcus	Ent	E. faecium DO	nc_017960.1	Human isolate
Enterococcus	Ent	E. faecalis ATCC29212	nz_cp008816.1	Human isolate
Enterococcus	Ent	E. faecalis CLB21560	nz_cp019512.1	Human isolate
Enterococcus	Ent	E. faecalis OG1RF	cp_017316.1	Human isolate
Enterococcus	Ent	E. faecalis DENG1	nz_cp0040801.1	Human isolate
Enterococcus	Ent	E. faecalis symbioflor1	cp_019770.1	Lab strain

Continued on next page

Table S1 – continued from previous page

Genus	Group	Strain	Accession number	Origin
Enterococcus	Ent	E. faecalis L12	nz_cp018102.1	Swine.
Enterococcus	Ent	E. faecalis L9	nz_cp018004.2	Swine
Enterococcus	Ent	E. faecalis D32	nc_018221.1	Swine
Enterococcus	Ent	E. faecalis KB1	nz_cp015410.2	Mouse
Enterococcus	Ent	E. faecalis sorialis	nz_cp015883.1	Mouse
Enterococcus	Ent	E. faecalis LD33	nz_cp014949.1	Included in dairy product
Citrobacter	KECS	C. koseri fdaargos287	cp_022073.1	UTI patient urine isolate
Enterobacter	KECS	E. cloacae atcc13047	nc_014121.1	Human isolate
Klensiella	KECS	K. pneumoniae kp36	nz_cp017385.1	UTI isolate
Klebsiella	KECS	K. pneumoniae hs11286	nc_016845.1	Reference strain
Klebsiella	KECS	K. oxytoca cav1335	nz_cp011618.1	Urine tract
Pantoea	KECS	P. ananatis lmg5342	nc_016816.1	Human isolate
Serratia	KECS	S. liquefaciens atcc27592	nc_021741.1	From a culture collection.
Morganella	MM	M. morganii kt	nc_020418.1	Human isolate
Proteus	Pm	P. mirabilis HI4320	nc_010554.1	Urine isolate
Proteus	Pm	P. mirabilis CYPM1	nz_cp012674.1	Urine isolate
Proteus	Pm	P. mirabilis BB2000	nc_022000.1	Human isolate
Proteus	Pm	P. mirabilis AOUC001	nz_cp015347.1	Human isolate
Pseudomonas	Ps	P. aeruginosa DK2	nc_018080.1	Human isolate
Pseudomonas	Ps	P. aeruginosa LESB58	nc_011770.1	Human isolate
Proteus	Pm	P. mirabilis AR0059	nz_cp020052.1	Human isolate
Proteus	Pm	P. mirabilis AR0155	nz_cp021694.1	Human isolate
Proteus	Pm	P. mirabilis AR0159	nz_cp021550.1	Human isolate
Proteus	Pm	P. mirabilis AR0156	nz_cp021852.1	Human isolate
Pseudomonas	Ps	P. aeruginosa PAO1	nc_002516.2	Human isolate.
Pseudomonas	Ps	P. aeruginosa SCV20265	nc_023149.1	Human isolate.
Pseudomonas	Ps	P. aeruginosa PA7	nc_009656.1	Human isolate.
Pseudomonas	Ps	P. aeruginosa YCBPP-PA14	nc_008463.1	Human isolate.
Pseudomonas	Ps	P. aeruginosa NCGM2-S1	nc_017549.1	Human isolate
Pseudomonas	Ps	P. fluorescens f113	nc_016830.1	Sugar-beet rhizosphere.
Staphylococcus	St	S. aureus mw2	nc_003923.1	R. of S. aureus
Staphylococcus	St	S. aureus Newman	nc_009641.1	R. of second group
Staphylococcus	St	S._haemolyticus_jcsc1435	nc_007168.1	Human isolate
Staphylococcus	St	S. haemolyticus Sh29-312-L2	cp_cp011116.1	Human isolate
Staphylococcus	St	S. haemolyticus S167	nz_cp013911.1	Leaf.
Staphylococcus	St	S. capitis ayp1020	nz_cp007601.1	Only one available
Staphylococcus	St	S. simulans fdaargos124	nz_cp014016.1	Only one available

Table S2. Table containing a second selection of genomes for the *in silico* UTI community. The information was retrieved in July 2021. Genomes were selected in a similar manner with the Supplementary Table 1

Genus	Group	Strain	Accession number	Origin
Escherichia	Ecoli	E.coli cft073	nc_004431.1	UPEC (Urinary)
Escherichia	Ecoli	E.coli 536	nc_008253.1	UPEC (Urinary)
Escherichia	Ecoli	E.coli IAI39	nc_011750.1	UPEC (Urinary)
Escherichia	Ecoli	E.coli UTI89	nc_007946.1	UTI isolate
Escherichia	Ecoli	E.coli E24377A	nc_009801.1	Human isolate
Escherichia	Ecoli	E.coli HS	nc_009800.1	Human isolate
Escherichia	Ecoli	E.coli O83-H1-NRG857C	nc_017634.1	Human isolate
Escherichia	Ecoli	E.coli O111-H-11128	nc_013364.1	Human isolate
Escherichia	Ecoli	E.coli O157-H7-Sakai	nc_002695.1	Human isolate
Escherichia	Ecoli	E.coli SE11DNA	nc_011415.1	Human isolate
Escherichia	Ecoli	E.coli O103-H2-12009	nc_013353.1	Human isolate
Escherichia	Ecoli	E.coli O104-H4-2011C3493	nc_018658.1	Human isolate
Escherichia	Ecoli	E.coli SE15DNA	nc_013654.1	Human isolate
Escherichia	Ecoli	E. coli EcPF5	cp_054236	UTI isolate
Escherichia	Ecoli	E. coli EcPF7	cp_054232	UTI isolate
Escherichia	Ecoli	E. coli EcPF14	cp_054230	UTI isolate
Escherichia	Ecoli	E. coli EcPF15	cp_054227	UTI isolate
Escherichia	Ecoli	E. coli EcPF16	cp_054224	UTI isolate
Escherichia	Ecoli	E. coli EcPF18	cp_054219	UTI isolate
Escherichia	Ecoli	E. coli EcPF40	cp_054214	UTI isolate
Enterococcus	Ent	E.faecalis FDAARGOS_338	cp_022059.1	Urine isolate
Enterococcus	Ent	E.faecium Aus004	nc_017022.1	Human isolate
Enterococcus	Ent	E.faecium Aus0085	nc_021994.1	Human isolate
Enterococcus	Ent	E.faecium DO	nc_017960.1	Human isolate
Enterococcus	Ent	E.faecalis ATCC29212	nz_cp008816.1	Human isolate
Enterococcus	Ent	E.faecalis CLB21560	nz_cp019512.1	Human isolate
Enterococcus	Ent	E.faecalis OG1RF	cp_017316.1	Human isolate
Enterococcus	Ent	E.faecalis DENG1	nz_cp0040801.1	Human isolate
Enterococcus	Ent	E. faecalis SF28073	nz_cp060804	Urine isolate
Enterococcus	Ent	E. faecalis TH4125	nz_cp051005.1	Urine isolate
Enterococcus	Ent	E. faecalis KUB3006	nz_ap018538.1	Urine isolate
Enterococcus	Ent	E. faecalis KUB3007	nz_ap018543.1	Urine isolate
Enterococcus	Ent	E. faecalis VE14089	nz_cp039296.1	Human isolate
Enterococcus	Ent	E. faecalis WE0438	nz_od940420.1	Urine isolate
Enterococcus	Ent	E. faecalis BX8117	nz_od940437.1	Urine isolate
Citrobacter	KECS	C.koseri FDAARGOS_287	cp_022073.1	UTI isolate
Enterobacter	KECS	E.cloacae ATCC13047	nc_014121.1	Human isolate

Continued on next page

Table S2 – continued from previous page

Genus	Group	Strain	Accession number	Origin
Klensiella	KECS	K.pneumoniae kp36	nz_cp017385.1	UTI isolate
Klebsiella	KECS	K. pneumoniae TOP52 1721	cp_031938	UTI isolate
Klebsiella	KECS	K.oxytoca cav1335	nz_cp011618.1	Urine isolate
Pantoea	KECS	P.ananatis lmg5342	nc_016816.1	Human isolate
Serratia	KECS	S. liquefaciens FDAARGOS_25	nz_cp014017	Human isolate
Morganella	MM	M.morganii KT	nc_020418.1	Human isolate
Proteus	Pm	P.mirabilis HI4320	nc_010554.1	Urine isolate
Proteus	Pm	P.mirabilis CYPM1	nz_cp012674.1	Urine isolate
Proteus	Pm	P.mirabilis BB2000	nc_022000.1	Human isolate
Proteus	Pm	P.mirabilis AOUC001	nz_cp015347.1	Human isolate
Proteus	Pm	P.mirabilis AR0059	nz_cp020052.1	Human isolate
Proteus	Pm	P.mirabilis AR0155	nz_cp021694.1	Human isolate
Proteus	Pm	P.mirabilis AR0159	nz_cp021550.1	Human isolate
Proteus	Pm	P.mirabilis AR0156	nz_cp021852.1	Human isolate
Pseudomonas	Ps	P.aeruginosa DK2	nc_018080.1	Human isolate
Pseudomonas	Ps	P.aeruginosa LESB58	nc_011770.1	Human isolate
Pseudomonas	Ps	P.aeruginosa PAO1	nc_002516.2	Human isolate
Pseudomonas	Ps	P.aeruginosa SCV20265	nc_023149.1	Human isolate
Pseudomonas	Ps	P.aeruginosa PA7	nc_009656.1	Human isolate
Pseudomonas	Ps	P.aeruginosa YCBPP-PA14	nc_008463.1	Human isolate
Pseudomonas	Ps	P.aeruginosa NCGM2-S1	nc_017549.1	Human isolate
Pseudomonas	Ps	P. fluorescens NCTC10783	nz_lr134300	Human isolate
Staphylococcus	St	S. aureus BLR-DV	nz_cp058312.1	Urine isolate
Staphylococcus	St	S. aureus USA300-SUR4	nz_cp014371	Urine isolate
Staphylococcus	St	S.haemolyticus jcsc1435	nc_007168.1	Human isolate
Staphylococcus	St	S.haemolyticus Sh29-312-L2	cp_cp011116.1	Human isolate
Staphylococcus	St	S. haemolyticus FDAARGOS_517	nz_cp033814.1	Human isolate
Staphylococcus	St	S.capitis ayp1020	nz_cp007601.1	Human isolate
Staphylococcus	St	S.simulans FDAARGOS_124	nz_cp014016.1	Human isolate

Table S3. Complementarity indices. All of them normalized by pathway length. D is the number of KO's present in the donor *d*; A is the equivalent for the acceptor *a*.

Name	Calculation
Complementarity 1	D - D∩A
Complementarity 2	(D∪A)/A
Complementarity 3	(D∪A-D∩A)/A
Complementarity 4	(D∪A – D∩A)/D∪A

Table S4. Competition indices. All of them normalized by pathway length. D is the number of KO's present in the donor d; A is the equivalent for the acceptor a. Competition 1 is only written as the intersection of two sets because stating a division by pathway length is omitted for all cases.

Name	Calculation
Competition 1	D∩A
Competition 2	(D∩A)/A
Competition 3	(D∩A)/D∪A
Competition 4	Pearson correlation / A

Table S5. Possible outcomes of the interactions depending on the value of ε.

Outcome	ε value
Positive effect on growth	$N_c > N_u(\varepsilon > ln(1.25) = 0.22)$
Neutral effect on growth	$N_c \approx N_u(ln(1.25) = 0.22 > \varepsilon > ln(0.8) = -0.22)$
Negative effect on growth due to competitiveness	$N_c < N_u(ln(0.8) = -0.22 > \varepsilon > ln(0.6) = -0.51)$
Negative effect on growth due to inhibition	$N_c << N_u(\varepsilon < ln(0.6) = -0.51)$

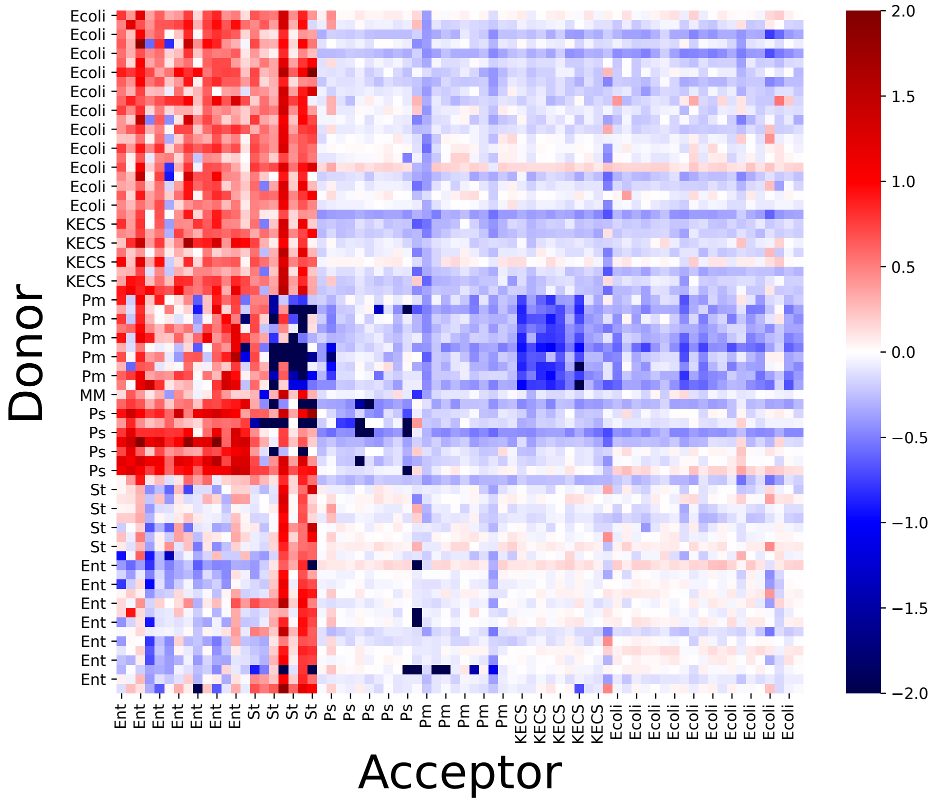

Figure S 1. Matrix representing the outcome of pairwise interactions, in terms of growth yield ($MaxOD_{600}$) achieved by 72 UTI isolates, in medium conditioned by these same isolates. The interaction index is $\varepsilon = ln(\frac{N_c}{N_u})$. The acceptors (columns) were grown in medium conditioned by the donors (rows). The interactions cluster together by phylogeny (16S phylogeny in [**deVos2017**], not shown here). Isolates are order symmetrically, and named by their group, from left to right: Ent (*Enterococcus spp.*), St (*Staphylococcus spp.*), Ps (*Pseudomonas spp.*), Pm (*P. mirabilis*), KECS (*Klebsiella spp.*, *E. cloacae*, *C. koseri*, *S. liquefaciens*, and *Pantoea sp4*), and Ecoli (*E. coli*). There is a *M. morganii* isolate between *Pseudomonas* and *P. mirabilis*. The diagonal from left-bottom to right-up represents self-interactions. Recreated based on [**deVos2017**].

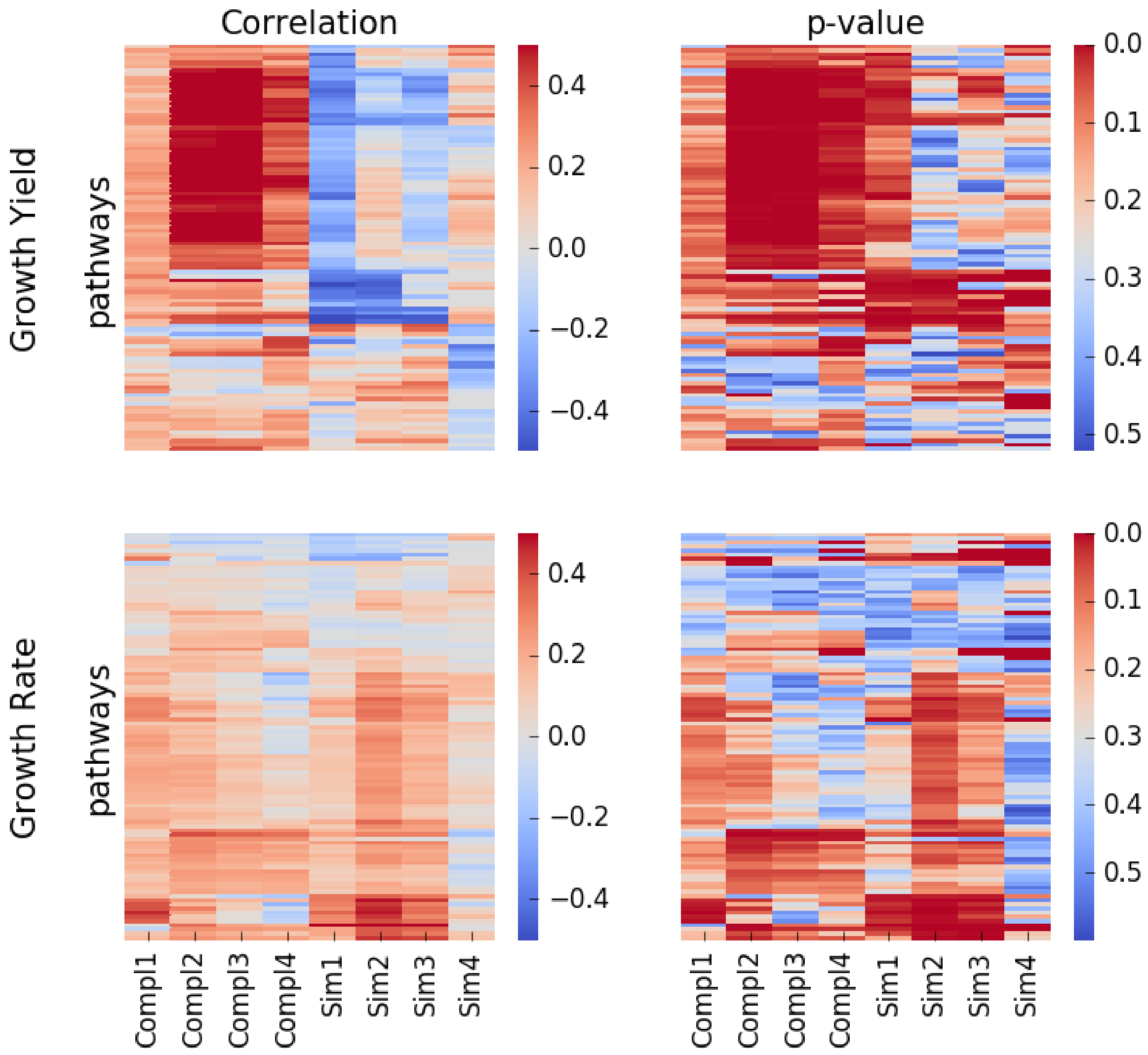

Figure S2. Correlation and corresponding p-values of different interaction measures. Correlation (left) and corresponding p-values (right) of different interaction measures with the values in the growth yield (up) and rate (bottom) pairwise matrices. Each row in the heatmaps represents a pathway; they are located in the same order. The measures complementarity 2 and complementarity 3 show the highest correlation, with a low p-value, for the data in growth yield. No measure correlates with the growth rate data.

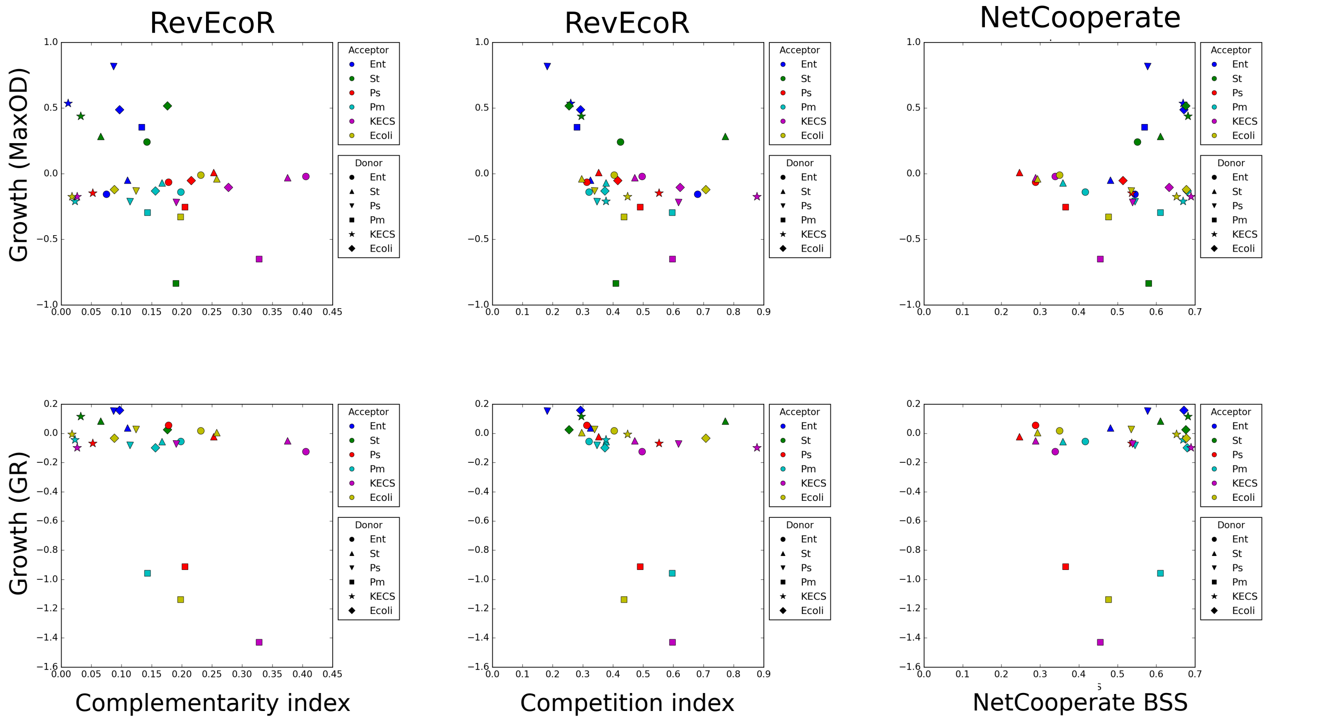

Figure S3. Relationship between RevEcoR and NetCooperate indexes and the experimental growth information. Interaction scatter plots depicting the relationship between the computed RevEcoR (complementarity and competition) and NetCooperate (BSS, Biosynthetic Support Score) indexes (x-axis) and the experimental growth information (y-axis). Each points represents the donor with a different shape, and the acceptor with a color; the values are grouped by the mean of the genera. Note that MM was omitted from the analysis community. The indexes are calculated for the complete set of KO's of every organism. In this case, a pattern could not be discerned.

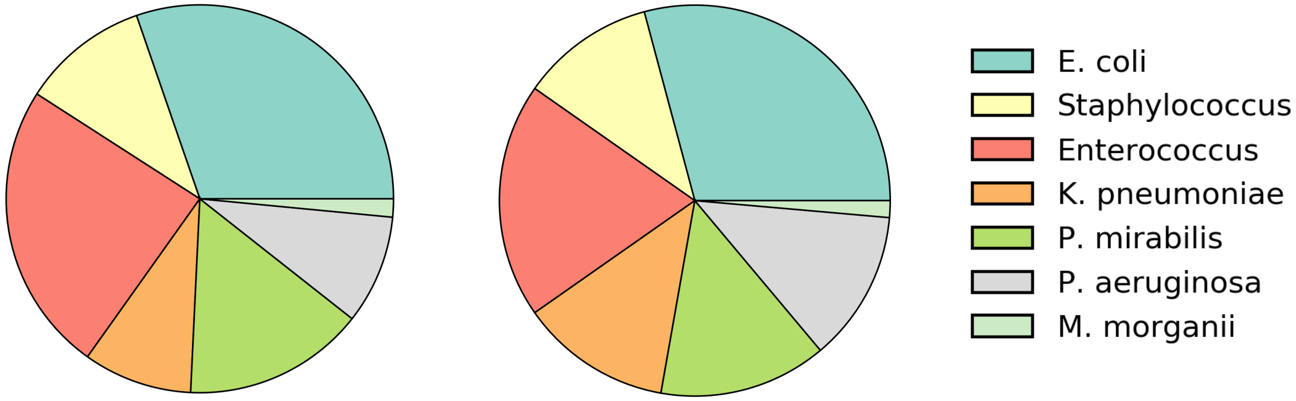

Figure S4. Composition of the UTI communities used for this work. The community built from 72 isolates from 23 patients (left), and the mimicking *in silico* community constructed from 66 related strains present in the NCBI database, include the same strains in a similar proportion. *E. coli* is the most abundant species, as it is present in many UTI patients.

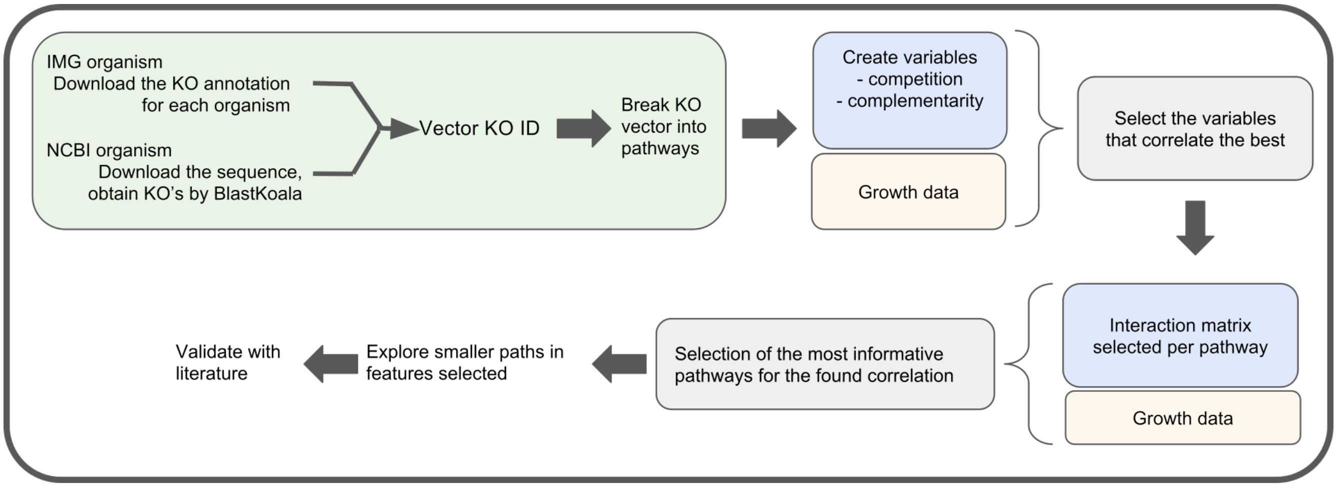

Figure S5. General flowchart. We started with a dataset of 66 genomes from NCBI. From them, the KEGG Ortholog (KO) annotation was retrieved from IMG database or using BlastKoala. The set of KO's obtained was then split into smaller units. We created variables related with microbial interactions that could be labeled as competition and complementarity. We then selected those who presented higher correlation with an initial pairwise interaction matrix that measured the effect in growth. The selected variables were used to select pathways that contribute to this correlation, again using the growth data. Then, they were studied visually using KEGG maps and hypothesis of interaction were created.

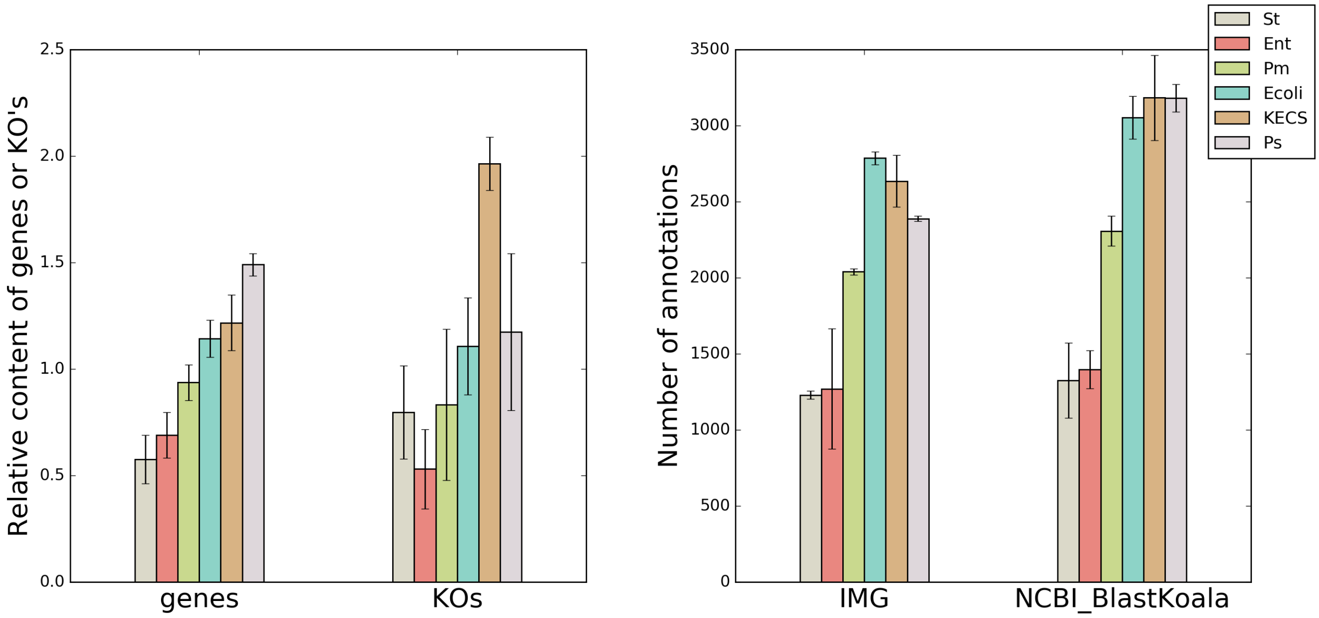

Figure S6. Comparison of the relative size of the genome and annotations per genera. (a) Comparison of the relative size of the genome (genes) and annotations (KO terms) per genera. Each of the bars depicts the relation between each genera's number of genes (left) or annotations (right), and the average for all genera. Interestingly, the difference in annotations for *KECS* is larger than the difference for genes, with more annotations than average for *KECS* and less annotations for enterococci. (b) Comparison of the number of annotations (KO terms) retrieved by two different approaches: NCBI genomes annotated with BlastKoala, and KO's directly downloaded from IMG. Overall retrieving the KO's using BlastKoala guarantees a higher coverage of the genome. *KECS* and *Pseudomonas* show the largest variation in the size of their KO set: 121% KO ID's obtained by the BlastKoala approach in *KECS* versus IMG, and 133% of the KO ID's in *Pseudomonas*.

Table S6. Similarly as Table 1 in the main text, but only on Ent as target. Pathways with high complementarity for the pairs with increased growth yield. Top ranking pathways according to the RF feature importance, the SVM coefficients, the MWU *p*-value, and the ensemble of the previous ranks.

Pathways	RF		SVM		MWU		Ensemble	
	**feat. imp.**	**rank**	**coef.**	**rank**	**p-values**	**rank**	**sum**	**rank**
Tyrosine metabolism	0.047	7	0.531	6	3.1E-05	1	14	1
Lipopolysaccharide biosynthesis	0.119	1	2.265	1	3.1E-04	13	15	2
Sulfur metabolism	0.052	6	0.888	3	2.7E-04	12	21	3
Cationic antimicrobial peptide (CAMP) resistance	0.043	9	0.513	9	1.4E-04	6	24	4
Phenylalanine metabolism	0.069	3	0.313	16	2.2E-04	10	29	5
Biotin metabolism	0.021	16	0.385	14	7.0E-05	4	34	6
Biofilm formation - Pseudomonas aeruginosa	0.056	5	0.318	15	7.2E-04	29	49	7
Bacterial secretion system	0.041	10	0.905	2	1.3E-03	41	53	8
Glutathione metabolism	0.005	28	0.243	22	9.4E-05	5	55	9
Riboflavin metabolism	0.014	19	0.518	8	6.6E-04	28	55	10
Folate biosynthesis	0.001	57	0.016	48	3.2E-04	15	120	30
Histidine metabolism	0	92	-0.107	65	3.2E-04	14	171	64

Table S7. Repetition of the Table 2, using the new selection of genomes in Table S2. Pathways with high complementarity for the pairs with increased growth yield.Top ranking pathways according to the RF feature importance, the SVM coefficients, the MWU p-value, and the ensemble of the previous ranks. The information was retrieved in July 2021.

Pathways	RF		SVM		MWU		Ensemble	
	**feat. imp.**	**rank**	**coef.**	**rank**	**p-values**	**rank**	**sum**	**rank**
Lipopolysaccharide biosynthesis	0.111	2	2.343	1	0.005	11	14	1
Biofilm formation - Pseudomonas aeruginosa	0.061	6	0.315	13	0.002	3	22	2
Cationic antimicrobial peptide (CAMP) resistance	0.066	5	0.329	11	0.003	6	22	3
Phenylalanine metabolism	0.081	3	0.227	21	0.003	4	28	4
Sulfur metabolism	0.041	7	1.31	2	0.008	20	29	5
Biotin metabolism	0.008	19	0.978	3	0.004	7	29	6
Tyrosine metabolism	0.008	20	0.325	12	0.002	1	33	7
Nicotinate and nicotinamide metabolism	0.029	13	0.295	14	0.006	13	40	8
Bacterial secretion system	0.036	10	0.653	6	0.019	31	47	9
Arginine biosynthesis	0.007	22	0.278	18	0.004	8	48	10
2-Oxocarboxylic acid metabolism	0.017	15	0.114	33	0.002	2	50	11
Glutathione metabolism	0.008	18	0.192	25	0.005	10	53	12
Histidine metabolism	0.04	9	0.099	34	0.007	15	58	13
Folate biosynthesis	0.001	69	0.033	45	0.045	51	165	56

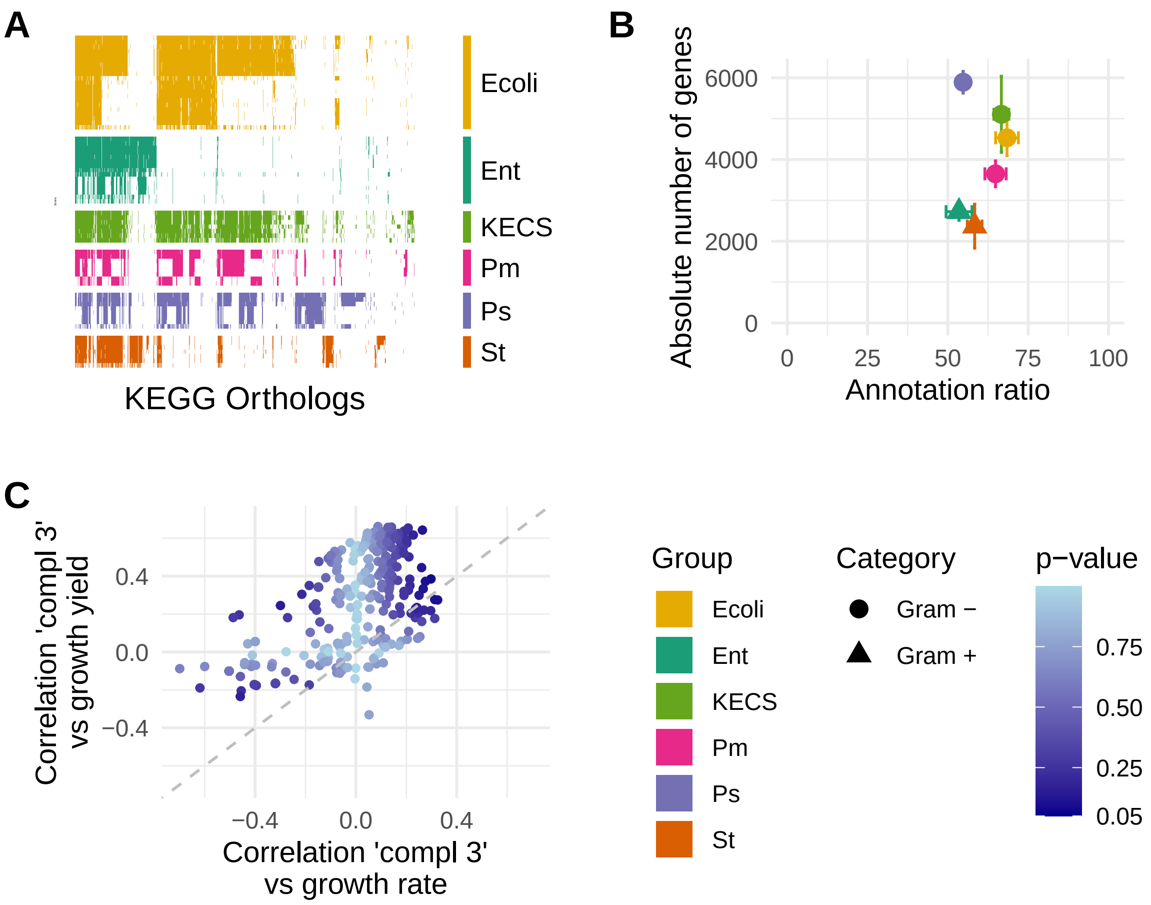

Figure S7. Repetition of the Figure 1, using the selection of genomes in Table S2. (**a**) Heatmap of presence (coloured) or absence (white) of KEGG Orthologs in the different bacteria groups. KEGG orthologs are grouped using a distance method relative to their Pearson correlation. The order of the KEGG orthologs on the horizontal axis therefore does not correspond to the order of Figure 1A in the main text. (**b**) While the absolute number of genes in the genome of each group varies per group, their annotation ratio from genome to KO's remains similar around 60%. (**c**) Comparison of the relation of 'complementarity 3' with the increase of two different growth measures: yield and rate (from [5]).
